# Machine Learning Techniques for Arousal Classification from Electrodermal Activity: A Systematic Review

**DOI:** 10.3390/s22228886

**Published:** 2022-11-17

**Authors:** Roberto Sánchez-Reolid, Francisco López de la Rosa, Daniel Sánchez-Reolid, María T. López, Antonio Fernández-Caballero

**Affiliations:** 1 Departamento de Sistemas Informáticos, Universidad de Castilla-La Mancha, 02071 Albacete, Spain; 2 Neurocognition and Emotion Unit, Instituto de Investigación en Informática, 02071 Albacete, Spain; 3 CIBERSAM-ISCIII (Biomedical Research Networking Center in Mental Health, Instituto de Salud Carlos III), 28016 Madrid, Spain

**Keywords:** electrodermal activity, arousal, machine learning, systematic review

## Abstract

This article introduces a systematic review on arousal classification based on electrodermal activity (EDA) and machine learning (ML). From a first set of 284 articles searched for in six scientific databases, fifty-nine were finally selected according to various criteria established. The systematic review has made it possible to analyse all the steps to which the EDA signals are subjected: acquisition, pre-processing, processing and feature extraction. Finally, all ML techniques applied to the features of these signals for arousal classification have been studied. It has been found that support vector machines and artificial neural networks stand out within the supervised learning methods given their high-performance values. In contrast, it has been shown that unsupervised learning is not present in the detection of arousal through EDA. This systematic review concludes that the use of EDA for the detection of arousal is widely spread, with particularly good results in classification with the ML methods found.

## 1. Introduction

Arousal is a general physiological and psychological activation of an organism, varying on a continuum from deep sleep to intense excitation. Performing a systematic review of arousal-related papers is challenging, as arousal encompasses a wide terminology. The construct arousal is a term that corresponds to the level of cortical activation that is regulated by the ascending reticular activation system. Arousal varies from a level of over-activation, as in the case of intense emotions or alert states, to a best attentional level for intentional action, or to levels of under-activation, as in the case of relaxation or sleep states. For example, the term stress is closely related to arousal in many works. Hence, it is possible to use the terms distress (negative stress) and eustress (positive stress) [1]. Another number of important papers study the change in arousal for the detection and classification of emotions. Indeed, according to Russel’s model of emotions, arousal is one of the variables that writes down the state of excitement towards a situation or event that provokes an emotional change [2]. In addition, variations in arousal are at the heart of experimenting with task-oriented activities such as driving [3] or figuring out mental workload at work.

There is a growing interest in developing methods for processing changes in arousal and using them in a variety of daily-living situations [4]. The most widely used technologies focus on the adoption of wearable devices. Such technologies usually work with the physiological conditions of the human body, using various variables to determine the activation state [5,6]. In fact, many researchers agree that variation in arousal correlates with increases in many physiological variables such as heart rate, electrodermal activity (EDA), breath intervals and skin temperature, among others [7,8]. Acquisition, processing and monitoring of physiological variables allow the creation of a map of the physical, mental and cognitive state of a subject [9,10]. Such a map is difficult to set up in many cases due to the origin of the physiological signals [11]. In any case, there are numerous physiological variables that are being used for arousal detection and its applications. We will focus on the analysis of EDA since it has been shown to be highly effective in the estimation of this excitement level.

EDA is considered especially useful in assessment of the arousal level due to its connection with the sympathetic nervous system (SNS) through the sudomotor system [12]. Alterations in the state of activation are unequivocally reflected as variations in skin perspiration, which affects the conductivity (conductance) of the skin. The measurement of these changes is excellent for estimating the psycho-physical state. In this respect, many causal models are used to infer sympathetic activation (arousal) from EDA signals such as curve fitting, inverse filtering, general linear model for evoked skin conductance response (SCR), non-negative deconvolution, continuous deconvolution, dynamic causal model (DCM) for anticipated SCR and DCM for spontaneous fluctuations [13].

We are not solely interested in EDA-based arousal detection in this systematic review, but the focus will be on the different machine learning (ML) methods used so far to classify excitement (arousal). Moreover, the review includes works using EDA alone or together with other physiological variables. Due to the substantial number of ML techniques and the proper nature of arousal, the present review is centred in classifying low versus high arousal (calm versus high excitement states), although considering both binary and multi-class methods. Moreover, given the diversity of the experiments found and the disparity in aims and design, our intention is to delve deeper into the possible connections among all the papers selected and to create a map of the most used techniques and their performance. In this sense, this review intends to create a conceptual map of the techniques used for EDA signal processing to help researchers find the best technique for processing such signals, allowing them to focus on fine tuning and optimisation of the different models. This map will contribute to the development of new processing and classification techniques.

The remainder of the article is as follows. Section 2 provides a brief explanation about the methods followed to perform the review. Section 3 introduces a summary on the status of the topic addressed in the review. Section 4 describes the most relevant results and a discussion about the studies found. Finally, Section 5 offers the conclusions of this work.

## 2. Review Protocol

### 2.1. Search Strategy

The reporting of this systematic review was guided by the standards of the Preferred Reporting Items for Systematic Review and Meta-Analysis (PRISMA) Statement [14]. A total of five scientific databases were selected for a wide search of ML and EDA in arousal detection. The selected databases were Scopus, IEEE Xplore, PubMed, ScienceDirect and ACM Digital Library. The selected papers were sought based on three distinct categories in the search criteria. The first focused on searching EDA-related terms like “skin conductance”, “electrodermal activity”, “galvanic skin response”. The second was centred on finding all the terms associated with arousal detection, such as “detection”, “identification” and “recognition” in conjunction with “stress”, “arousal”, “activation”, “agitation”, “excitement”, “emotion”, “mental workload”, “cognitive workload” and “pain” terms. Finally, the third term that completed the search chain aimed to look for classification methods in the field of Artificial Intelligence: “machine learning” and “deep learning”. The systematic review was conducted from the time records are kept in each of the databases until June 2022.

The consultations were refined by successive searches to get as small a set of search terms as possible without losing the scope of the review. This allowed us to keep a manageable number of keywords without losing the perspective and focus of the systematic review. A series of inclusion and exclusion criteria were established to filter the desired information:

- Inclusion criteria
  – Publications implementing and evaluating the performance of ML-based methods and algorithms for low/high arousal level detection, identification and recognition using EDA as basis.
  – Articles written in English.

- Exclusion criteria
  – Literature with an unclear peer review process (grey literature): tutorials, toolkits, editorials, extended abstracts, PhD symposium papers, keynotes, research summaries and technical reports.
  – Systematic reviews (including meta-analyses) and survey documents.
  – Conference papers and book chapters.
  – Articles published after 30 June 2022.
  – Articles posted on a preprint database.

**Figure 1:**
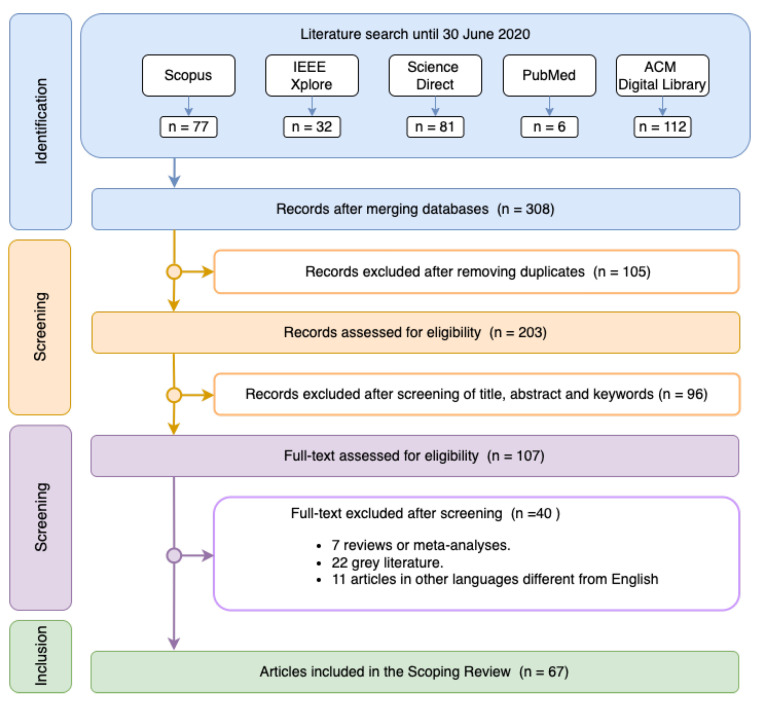
Search strategy.

Figure 1 details the scheme followed to obtain the final selection of the articles in the systematic review. The *identification* stage resulted in a total of 308 papers, of which 77 papers were obtained in Scopus, 32 in IEEE Xplore, 81 in ScienceDirect, 6 in PubMed and 112 in ACM Digital Library. The papers were selected and eliminated according to the inclusion and exclusion criteria mentioned above during the *screening* stage. A total of 105 duplicates were removed from the various databases. In addition, 88 articles were removed after reading their abstract as they were outside the scope of the review. The criterion was to select papers that used EDA signals alone or together with other signals and employing ML techniques. Finally, 40 articles from the remaining 107 articles were removed in the last stage (*inclusion*) after a thorough reading of the complete content. This way, 67 articles were left for study in this systematic review.

### 2.2. Paper Classification Categories

Two categories were proposed once all the articles had been examined. The first, shown in Table 1, classified the papers based on their scope of coverage in six groups: arousal, stress, emotion, physical pain, task-oriented and others. The group arousal focuses on those papers that deal with the detection, processing and usage of the EDA signals to determine the arousal level. Stress is centred on articles concerned with the detection and classification of some stress-inducing situations. The emotion group focuses on papers related to any aspect of detection and classification of emotional states. Another group of papers is related to physical pain detection. A fifth group (task-oriented) is dedicated to studies on changes in arousal when performing a single-task-oriented procedure such as driving a car. A sixth category refers to mental or cognitive workload. Lastly, the other classes stand for monitoring other human body states such as sleep and dehydration.

**Figure 2:**
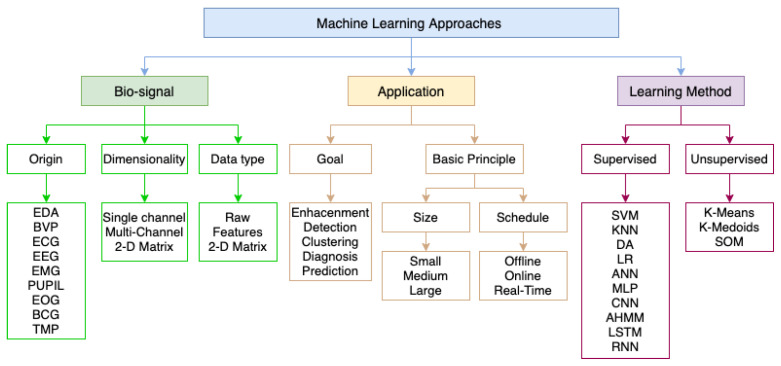
Paper grouping.

The second categorisation is shown in Figure 2. The first resulting category, *Biosignal*, is grounded on the different bio-markers used for obtaining the arousal level. The specific bio-signals for the detection of arousal are presented. Dimensionality of the data source is also identified, i.e., whether a sole source or multiple indicators are used for detection. In addition, the type of data used for detection is provided, differentiating between raw data, processed data and two-dimensional matrix. The second category, *Application*, focuses on applications that employ diverse types of classifiers intended for a specific use. It centres on the goals to be achieved, focusing on the creation of applications for the detection, grouping, diagnosis and future prediction of arousal. Other basic classification principles are whether the application runs with a large or small number of participants and signals and whether the system is used offline or in real time. This category is not dealt with in depth in this article, as it falls beyond the scope of this paper. The last category, *Learning Method*, is focused on the use and relevance of different learning methods for the detection task. Most analysed works base their learning ability on supervised classification algorithms, while the use of unsupervised classifiers is minor.

## 3. Methods on Arousal Detection

The human body may be regarded as an electromechanical system composed of perceptual, affective and cognitive processes. Its dynamic changes allow one to take different measurements on various bio-signals. The temporal signals make it possible to establish the physical, psychological and cognitive state of the human being with adequate precision [88,89]. Most biological signals involve electrical activity and conductivity along with changes in flow, temperature, volume, pressure, sound and acceleration [60,90,91,92].

There are many physiological variables which can be collected from the human body. The most common are the following. (a) The electrocardiogram (ECG) measures any change in heartbeat and pattern of beating [93,94]. (b) Electromyography (EMG) monitors changes in neuromuscular activity. (c) Blood volume pressure (BVP) measures changes in blood volume, which affects blood pressure by changing the cardiac output. (d) Electrooculography (EOG) allows monitoring of eye movements. (e) Pupillography or pupillometry (PUP) is based on the measurement of the pupil diameters under basal conditions and after applying different stimuli. (f) Electroencephalography (EEG) measures the variation of electrical signals produced in different areas of the brain. (g) Inter-breath (IBR) measures the rate of breathing. (h) Acceleration (ACC) monitors body movements. (i) Skin temperature (TMP) is used to quantify temperature variations. (j) Electrodermal activity (EDA) is used to check the arousal, this being an important variable for measuring the emotional state of a person. Table 2 describes the main properties of those bio-markers.

### 3.1. Signal Acquisition and Processing

**Figure 3:**
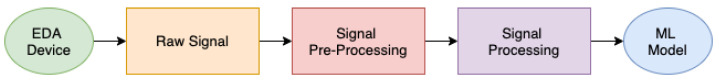
Usual stages in signal acquisition, pre-processing and processing.

Signal acquisition is one of the most important stages when using EDA (or any other bio-signal). Most authors referenced in this systematic review agree that a good acquisition process is crucial for the proper functioning of the later recognition system. Figure 3 shows the usual pathway for signal treatment. Here, the first stage is the acquisition of the raw signals by the EDA device. The next stage is pre-processing, which eliminates all the defects that have caused interference during the acquisition process. As part of this operation, artefacts are removed and the signal is filtered, making it softer and eliminating noise. The last stage is signal processing, where a series of features of the signal are obtained as a rule. ML models will later use these features.

#### 3.1.1. Raw Signal Acquisition: Datasets and Experimental Design

**Figure 4:**
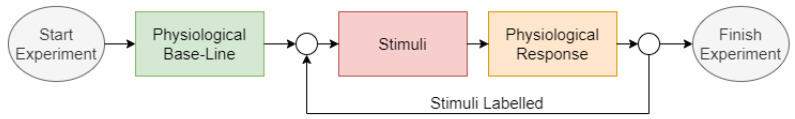
Flowchart of the experimental design during raw signal acquisition.

According to the outcomes of our systematic review, the authors always choose between two different procedures to acquire the raw signals. The first one is to create an experimental design as shown in Figure 4. A first step is to start the experiment; then begins the physiological baseline recording of the input data. Next, the person is subjected to a sensory stimulus, most commonly visual and auditory and the individual’s reactions are recorded. These stimuli trigger an autonomic response in the different systems [95,96]. The process is repeated as many times as necessary.

An alternative procedure uses several datasets already validated by the scientific community. These datasets usually hold a number of other physiological signals registered in addition to the EDA signal for use in multi-class classifiers. The most common datasets for EDA analysis are MAHNOB [97], DEAP [98], BioVid [66] and UT Dallas Database [99].

#### 3.1.2. Signal Pre-Processing: Normalisation, Artefact Removal and Noise Filtering

Pre-processing cleans, adapts and prepares the signals for further processing. This process is also fundamental to many authors who agree that the effectiveness of a classification system starts at this stage. Usually, pre-processing includes three different steps: signal normalisation, detection and elimination of artefacts and filtering of noise.

The first step aims at eliminating the subject-dependent baseline. This is done to reduce the amplitude of the variance [71,100,101,102]. Then, artefacts that interfere with the signal must be removed. A motion artefact (MAt) degrades signals very quickly and makes them unusable [23]. Artefacts are eliminated by deflecting the signal through various softening filters [103,104]. This procedure causes in most cases a loss of information in EDA signals. In addition, MAt detection consists of identifying each of the signal segments where the artefact removes it at later stages [22,23]. Noise reduction or elimination is strongly associated with the artefact detection and/or removal process. The most worrying noise in EDA signals is the high-frequency noise due to its slow evolution [92]. Therefore, the EDA signals are filtered to remove artefacts and noise recorded during the acquisition period. Two distinct types of filters are usually used; firstly, a low pass filter with a 4 Hz cut-off frequency and secondly, a Gaussian filter to attenuate the signals, artefacts and noise.

#### 3.1.3. Signal Processing: EDA Deconvolution

The measurement of EDA signals is usually conducted in two separate ways. The first manner is the *exosomatic* one, which is obtained from the variation of the resistance or conductance by injecting a small current into the skin. The second way, the *endosomatic*, is obtained from the measurement of the potential [105]. These measurements are composed of the convolution of two signals: a first signal that varies slowly, called the electrodermal level (EDL) and a second signal that varies rapidly, the electrodermal response (EDR). The EDL signal sets up the base level of the signal while the EDR is closely related to the activity of the sweat motor system, which is strongly associated with the sympathetic nervous system at the same time [106].

**Figure 5:**
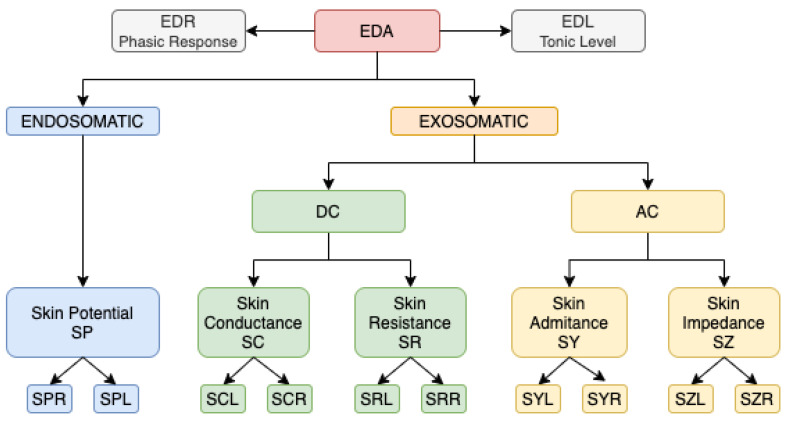
Contemporary labelling of electrodermal activity, inspired in [105].

Figure 5 sheds light on this division. In the endosomatic measurement lies the skin potential (SP), which, in turn, is divided into the skin potential response (SPR) as a phasic response and the skin potential level (SPL) as a baseline. On the other hand, exosomatic measurement is composed of two groups, AC and DC, depending on whether alternating or direct current is injected into the skin between the electrodes. For the EDR we have variables SCR, SRR, SYR or SZR related to conductance, resistance, admittance and impedance, while the variables SCL, SRL, SYL and SZL are used to evaluate the EDL.

**Figure 6:**
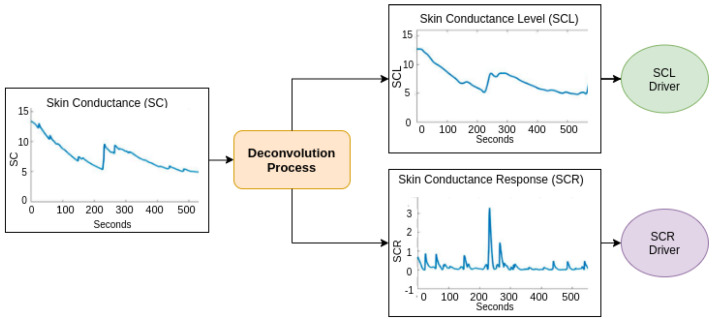
Flowchart of the deconvolution process.

The deconvolution procedure consists of separating the EDR signal from the EDL. This process minimises external effects such as temperature and humidity on each participant’s baseline. It also mitigates the effects of gender, race, physical condition and age of the participant [107,108,109]. In this sense, it normalises the signal so that the EDR is used as a common indicator for all the participants who have undergone the same stimulus. A process of deconvolution/decomposition is needed to obtain the components needed both for endosomatic and exosomatic measurements. Figure 6 illustrates the deconvolution process of the skin conductance (SC). As can be seen in the figure, the SCR driver is used to detect the level of excitation of the individual.

Mathematically, the sudomotor nerve function may be considered a driver with a train of impulses that evolve over time. This response is embedded in the *SCR* and *SCL* signals [110,111]. The outcome is presented by a convolution (“∗” symbol) of the driver with the impulse-response function (*IRF*), describing the impulse response flowing through time as shown in Equation (1).

(1) $$SC=SC_{Driver}*IRF$$

The SC signal is formed by the SCL and SCR signals, as displayed in Equation (2).

(2) $$SC=SCL+SCR=SCL_{Driver}*IRF+SCR_{Driver}*IRF$$

(3) $$SC=(SCL_{Driver}+SCR_{Driver})*IRF$$

Thus, the tonic signal driver is obtained by deconvolution (“/” symbol) of Equation (3) as:

(4) $$SC/IRF=SC_{Driver}=SCL_{Driver}+SCR_{Driver}$$

The process can be conducted in two manners. The first, the *continuous decomposition analysis*, decomposes *SC* data in continuous tonic and phasic activity. This approach, which is based on standard deconvolution, is fast and robust against artefacts. The second is *discrete decomposition analysis*, which separates the SC data in a tonic component and discrete phasic components with a no-negative deconvolution. This strategy captures and explores all deviations of the final response form and computes an in-depth full model of all parts within the entire dataset [92,111].

Many authors agree that deconvolution produces a normalisation in the signal, allowing to compare between different captured signals and subjects [49,112].

#### 3.1.4. Other EDA Processing Techniques

Although most of the articles found in the reviewed literature refer to the deconvolution process, there are other techniques that are used for EDA signal processing. Here we will mention some of them.

##### Complex Optimisation on EDA Signals (cvxEDA)

A novel algorithm for the analysis of EDA signals uses convex optimisation methods. EDA is one of the most widely observed pathways of sympathetic nervous system activity and is expressed as a change in the electrical properties in skin conductance (SC) [17,113]. This model represents the SC as the composite of three terms: the phasic component, the tonic component and an additive white Gaussian noise that incorporates the model’s prediction errors as well as measurement errors and artefacts. The model is physiologically inspired and fully explains EDA using a rigorous method based on Bayesian statistics, convex mathematical optimisation and sparsity. One benefit of this method is its low computational cost and that it can be incorporated into a variety of wearable devices.

##### Sparse Deconvolution Approach (sparsEDA)

Staying with models that have a low computational cost, the sparse deconvolution-based method called sparsEDA should be mentioned. This fully automated method was proposed for tonic/phase decomposition of EDA data based on non-negative sparse deconvolution and multi-scale modelling of SCRs. This method aims to strike a balance between filtering noise and improving the relevant insights into the EDA signals [113,114]. This lightweight method can also be embedded in a wearable device.

##### Spectral Analysis on EDA Signals

Spectral analysis is another novel approach for signal processing, motivated in part by advances in the analysis of heart variability (HRV) [115]. This method evaluates the dynamics of the autonomic nervous system by calculating the power spectrum in two main bands, a low frequency band corresponding to the limits [0.08–0.24] Hz and a high frequency band corresponding to the limits [0.25–0.4] Hz. The peak of maximum activity would be around 0.34 Hz for a high arousal activation zone [113]. As this procedure is inspired by the spectral analysis of the HRV, the low frequency band is thought to be related to the activation of the sympathetic and parasympathetic systems, while the upper band is only due to the influence of the parasympathetic system.

##### Cepstrum Analysis (CA)

This is the discrete-time inverse Fourier transform of the logarithm of the magnitude (*X*) of the discrete-time Fourier transform (DTFT) of the signal. It is formulated as:

(5) $$c\left[n\right]=\frac{1}{2\pi }\int_{-\pi }^{+\pi }log\left|(X\left(e^{i\omega }\right)\right|e^{i\omega n}d\omega$$

where $e^{i\omega }$ is the DTFT of the signal [86]. CA has successfully been used to isolate the basic waveform and the excitation function of physiological signals such as EDA [71], EEG [116] and ECG [117]. CA might be helpful for analysing overlapping EDA signals given its ability to amplify small amplitude variations. This analysis yields a series of coefficients called Mel-frequency cepstral coefficients (MFCCs) that are used as features introduced into the classification system (see Equation (5)).

##### Entropy Analysis (EA)

This describes the randomness, uniformity and disorder of a given system. Many features of the entropy domain have been used to analyse EDA signals [118]. EA allows us to detect patterns in the signal by using Shannon entropy [119]:

(6) $$H=-\frac{1}{logN}\sum p_{i}log\left(p_{i}\right)$$

where *N* is the number of observed events and $p_{i}$ is the probability that the *i*-th event occurs. Since Shannon entropy values differ with respect to the acquired data, it may be used as a feature to measure the characteristics of a signal (see Equation (6)).

##### Identification of the Dynamics of the Autonomous System

This approach consists of showing the dynamics of the autonomic system across different stimuli exposures [120]. For this purpose, several features are extracted from the EDA signals. A logistic regression (LOC) or receiver operating characteristic (ROC) process is then applied. These indices are concatenated for the different time windows of the signals that will later be processed by the LASSO regulation algorithm. Not all features survive this process, but the remaining ones supply much information about the condition of the participant. This allows for comparison in relation to the different situations or stimuli to which he/she has been exposed.

##### Models to Extract Pulses from EDA Signals

A systematic and robust approach to extract pulses from EDA data that preserve the statistical structure of physiologically derived data while excluding the noise has been developed [121]. This method exploits a total of seven parameters through four models (inverse Gaussian, log-normal, gamma and exponential) to figure out how to extract pulses. These pulses allow an assessment of the signal-to-noise profile of an entire data companion and the identification of individual subjects. From this emerges a line of analysis that is computationally accurate, statistically rigorous and physiologically based.

##### Poral Valve Model

This model favours the functioning of the activation of the autonomic system to produce a change of sweating in the skin. So, it models very efficiently the functioning of the different pores of the skin and its sweat activation, adopting a physiological approach to determine the different stages of activation or arousal produced [122].

#### 3.1.5. Feature Extraction

Feature extraction is usually performed using specially designed frameworks and methods. The most used frameworks are Ledalab [92] and cvxEDA [17] and the SparseEDA [112,114] method. Five main groups of features are distinguished: *time domain features* which refer to all the variables defined in terms of time; *frequency domain features* which refer to all the parameters defined in or based on frequency; *statistical features* defined as variables that belong to the statistical field; *morphological features* that quantify the shape of the signal; time-frequency features that characterise the signal in time and frequency domains simultaneously. Table 3 shows several features that usually characterise the different segments of SP, SC, as well as their tonic and phasic components (SPL, SPR, SCL and SCR). It should be noted that these features are used to characterise the signals more accurately. It is a good practice to use the best features that are most suited in relation to their contrasting performance.

The following features are commonly used in the time domain: mean amplitude (Mean); amplitude standard deviation (SD), the SD first and second derivative (D1, D2), the SD means (D1M, D2M) and their standard deviations (D1SD and D2SD) [26]; sum rise time (SRT), sum fall time (SFT), rise rate mean (RM), rise rate standard deviation (RRSTD); decay rate mean (DCRM), decay rate standard deviation (DCRSD); phasic value mean (PHVM), phasic value standard deviation (PHVSD); startle time mean (STM), startle time standard deviation (STSD), startle RMS mean (STRMS), startle RMS standard deviation (STRMSSD); startle RMS overall (STRMSOV); electrodermal level (EDL), electrodermal response (EDR); cumulative maximum (CMax), cumulative minimum (CMin); smallest window elements (SWE); dynamic range (DR); root-mean square level (RMS), peak-magnitude-to-RMS ratio (PMRMSR); root-sum-of-squares level (RSSL); peak (P), peak location (PLoc), peak to peak time (PPT), analysis of peaks with a time difference of more than 50 ms (pNN50) [25,29,46,47,65,69].

Distinctive features are available following the morphology of the signals: epoch-capacity (EC) is a relation between the number of epochs and the total number of them; epoch-peak (EP); epoch peak counter (EPC) is a number of epochs in all times; entropy (EN) [80]. On the other hand, there are features that result from different measurements such as arc length (AL), integral area (IN), normalised mean power (AP), root mean square (RMS), perimeter to area ratio (IL) and energy to perimeter ratio (EL) [26]. These parameters are due to the need to understand the morphological differences in the shape of the $SCR_{Driver}$. As far as statistical parameters are concerned, let us highlight mean value (M), variance (Var), median value (MedVal), *p*-value (p-Val), Akaike information criterion (AKAIKE), Log-likelihood (LOG-LIKE), covariance matrix (COVMAT), transition probabilities lag (TPL), number of observations (NO), switching betas (beta-Numb), number of estimated parameters (STP), standard error coefficient (SCE), smoothed probabilities of regimes (SPR), conditional standard deviation (CSTD), four central moment (FCM), five central moments (FVCM), kurtosis (KU), skewness (SKU) and momentum (MO) [59,69].

The following parameters are usually found in the frequency domain: sum spectral components (SSP), spectral power (SP), mean and spectral components (MSSP and SSPMed, respectively), frequency non-specific of skin conductance response (NSSCRs) and fast Fourier transform (FFT) for bandwidths F1 (0.1, 0.2), F2 (0.2, 0.3) and F3 (0.3, 0.4) [26,59,69,123,124,125]. Frequency bands with ranges [0.02–0.25 Hz], [0.25–0.40 Hz] and [0.40–1 Hz] have also been used as a measure of power spectral density (PSD) [113,126].

Finally, for time-frequency features, STFT is a basic principle for characterising the signal simultaneously in both domains. It is an application of the conventional fast Fourier transform applied to successive data segments using a short-time window. The time-frequency flux measure ($TF_{Flux}$), the time-frequency flatness measure ($TF_{Flatness}$), the time-frequency energy measure ($TF_{Energy}$) and the mean of time-varying spectral amplitudes in frequency bands (TVSymp) [127] use this approach. Mel-frequency cepstral coefficients (MFCCs) were included to quantify the EDA signals. Lastly, Shannon entropy ($E_{Shannon}$) and its logarithmic representation ($E_{Log}$) [49,128] have been found for entropy measures.

### 3.2. Machine Learning for Arousal Classification

As a rule, signal-based experiments yield a large number of extracted features to classify. ML techniques are used more than purely statistical ones to classify such enormous amount of data. Therefore, a comprehension of existing ML models, their main characteristics and methods of evaluation and their most relevant results is essential.

#### Evaluation Metrics

According to the literature studied, stress detection, physical pain detection, dehydration sensing and sleep monitoring are limited to a binary classification problem, while multi-class classifiers have been used for emotion detection and task-oriented applications. The different metrics that have been employed to measure performance are the following:

- Accuracy (ACR): degree of closeness to true value. In terms of TP (true positives), TN (true negatives), FP (false positives) and FN (false negatives):
  (7) $$\mathrm{ACR}=\frac{TP+TN}{TP+TN+FP+FN}$$
- Precision (P): ratio of successful positive predictions.
  (8) $$P=\frac{TP}{TP+FP}$$
- Recall (R) or Sensitivity (Se): fraction of relevant instances retrieved.
  (9) $$R=\mathrm{Se}=\frac{TP}{TP+FN}$$
- Specificity (Sp) or true negative rate (TNR): proportion of negatives that are correctly identified.
  (10) $$\mathrm{Sp}=\mathrm{TNR}=\frac{TN}{TN+FP}$$
  (11) TNR+FPR=1
- False positive rate (FPR): proportion of negative cases incorrectly identified as positive cases in the data.
  (12) $$\mathrm{FPR}=\frac{FP}{FP+TN}$$
- F1-score or F-measure: harmonic mean between precision and recall.
  (13) $$F1-\mathrm{score}=\frac{2\times P\times R}{P+R}\times 100$$
- Area under the curve (AUC) and receiver operating characteristics (ROC) curve: performance measurements for classification problems at various threshold settings.
- Precision-recall (PR) curve: this summarises the trade-off between the TPR and the positive predictive value for a predictive model using different probability thresholds.
- Confusion matrix (CM): a specific table disposition that allows one to visualise the performance of an algorithm.
- Cohen’s kappa-coefficient (κ): this is a measure of how closely the instances classified by the ML classifier match the data labelled as ground truth.
  (14) $$\kappa =\frac{{\mathrm{ACR}}_{0}-{\mathrm{ACR}}_{e}}{1-{\mathrm{ACR}}_{e}}$$
- Youden’s index (*J*): this is used to measure the sensitivity of each classifier.
  (15) J=Se+Sp−1

### 3.3. Classification Methods

Different classification methods have been found in the papers analysed in this systematic review. These methods can be grouped in relation to distinct categories. In the first place, there is *direct classification* vs. *hierarchical classification*. Furthermore, there is *long-term* vs. *short-term* when considering the duration of the classification. Finally, we can distinguish between *supervised* and *unsupervised learning* methods. Another aspect that must be considered is that ML models have some limitations due to the substantial number of parameters managed. Consequently, it is necessary to know how to implement methods that help us to reduce the number of redundant or irrelevant parameters. Therefore, dimensionality reduction techniques are becoming significant in the areas of ML, data mining and bioinformatics.

The feature reduction methods detailed next are usual to signal processing. Principal component analysis (PCA) is a standard statistical data analysis which tries to explain observable signals as a linear mixture of the orthogonal principal component that optimises the variance between the different components. Linear discriminant analysis (LDA) is typically used to reduce the dimensionality by maximising the space between the different classes. Finally, independent component analysis (ICA) is an analysis and data processing strategy that recovers unobservable signals or sources of monitored mixtures only under the assumption of mutual independence. These feature reduction techniques allow the leverage the computational cost since the resulting classifier is simpler and only attends to the key features of the signal. Many of the papers studied in this overview use such techniques and the results are really good compared to others that do not use them. Below, there is an explanation of the different methods used.

#### 3.3.1. Direct vs. Hierarchical Classification

We found direct and hierarchical classification methods in many articles analysed in this review. A *direct classification* consists in classifying the arousal of the person in a direct way considering one or more physiological variables. On the other hand, there are two distinct stages when a *hierarchical classification* is proposed. The arousal is established in a first stage and a more complex emotional state can be classified in a second stage [59].

#### 3.3.2. Long-Term vs. Short-Term Affective State Classification

Whether a classification of the emotional state should consider the duration of the experiment as well as the evolution of the signals over time are other aspects to be considered. The first issue to highlight is the need for a classifier that works quickly and is consistently robust over a long period. In this sense, a classification could be defined as *short-term* or *long-term*. The former is aimed at instantaneously finding results, while the latter is oriented towards long-term applications. A long-term classification is usually recommended in the context of stress detection [26].

### 3.4. Supervised vs. Unsupervised Learning

Within the different learning methodologies, there are (apart from reinforcement learning and stochastic learning) two other main groups, namely *supervised* and *unsupervised* learning [129].

#### 3.4.1. Supervised Learning Methods

Supervised learning techniques are based on training a classifier from a dataset that is already labelled. Once the system has learned to identify the different patterns, the classifier is able to effectively distinguish between the different classes. In our case, it must distinguish between low and high arousal, calm and stress and so on. There is a wide range of classifiers with supervised learning found in the papers selected:

- Support vector machines (SVMs) [130,131]. From the point of view of arousal detection from EDA, this is one of the most used algorithms, more concretely using *linear* [29,30,43,65], *quadratic* [29,46,71], *polynomial* [29,30,46], *Gaussian* [29,30] and *radial* [15,18,22,23,25,30,31,42,43,44,45,47,48,49,52,53,54,55,58,61,69,71,73,74,75,79,132,133] kernels.
- Auto-hidden Markov models (AHMMs) [57,59]. Different approaches have been used to find the status of each person from the EDA signals using AHMM [57,59].
- Discriminant analysis (DA). There are many classifiers based on DA, with the most common for the detection of arousal in EDA being: *linear discriminant analysis* (LDA) [25,70]; *quadratic discriminant analysis* (QDA) [27,30,49,52,81] and *Gaussian discriminant analysis* (GDA) [29].
- Decision trees (DTs) [134]. Within this type of classifier, the most used for arousal detection are *tree medium, regression tree* [27,42,45,61,80,81] and other ensemble methods like *random forest* and *bagged tree* [46,80].
- Naive Bayes. In this study, it has been found that the most used naive Bayes methods are *naive–Bayes–Gaussian* [42,44,52,61,80] and *naive–Bayes–Gaussian with PCA* [61,80].
- Logistic regression (LR). According to the references found, different papers have been published where this method is used as *logistic regression* [23,27,48,79] and a variant called *zero-regression* [48].
- A K-nearest neighbours (KNN) [135]. Within the different configurations that have been found are *KNN-Fuzzy* [46], *KNN-Fine* [46], *KNN-Cubic* [46,70], *KNN- Medium* [25,27,42,44,45,47,54,57,69,79] and *KNN-Weighted* [23].
- Artificial neural networks (ANNs). It should be noted that there are many topologies that have been used for the processing of the obtained features, such as *feed-forward NN* [69], *multi-layer perceptron with back-propagation* (MLP) [23,27,43,61,67,75,81], *Bayesian probabilistic NN* (BPNN) [44], *probabilistic NN* [61], *one-dimensional convolutional NN* (1D-CNN) [69,70] and, finally, *convolutional NN* (CNN) [15,44,49,53,71,73].
- Long short-term memory (LSTM) and recurrent neural networks (RNNs) [136,137]. In this systematic review, *LSTM* [34], ensemble-based methods like *CNN + LSTM* [34] and adaptive neurofuzzy inference system *(ANFIS-based short-term)* [25] have been used.

#### 3.4.2. Unsupervised Learning Methods

The second group of learning methods addressed is unsupervised learning [138]. This type of methods is based on learning by using an unlabelled dataset. The model obtained is automatically adapted to the observations. The model is created with clustering methods. According to the literature found in the systematic review the following unsupervised methods have been used:

- K-means is a clustering method, aimed at splitting an unlabelled dataset of *n* observations into *k* groups in which every single observation belongs to the group whose mean value is the closest [47].
- K-medoids is a grouping approach for the partitioning of a dataset into *k* groups or *k*-clusters, each group being represented by one of the group data points called cluster medoids [47].
- A self-organising map (SOM) is a type of ANN that is formed by the use of unsupervised learning to generate a low-dimensional map, typically two-dimensional [139]. In the selected literature we have found the use of SOMs for the detection of arousal [47,52].

## 4. Results

This section presents the different results obtained along this systematic review. Different analyses of the data obtained are conducted in this type of review as has been mentioned throughout the paper. Firstly, papers have been grouped according to physiological variables used for the determination of arousal. A second analysis focuses on determining which are the most typical classifiers (supervised and unsupervised) for arousal detection. For this purpose, the different classification methods have been grouped according to their similar configurations or topologies. In this way, estimating the most common ML technique is possible through concentrating the efforts on selecting a firm configuration and discarding those techniques that are known beforehand to perform poorly.

### 4.1. Bio-Markers Used in the Papers

One of the considerations taken during this study was to analyse the number of articles that only use the EDA to perform the different classifications. In addition, we are interested in those in which other bio-markers are used in conjunction with EDA to strengthen the classification results. As can be seen in Table 4, Table 5, Table 6, Table 7, Table 8, Table 9 and Table 10, the publications have been grouped according to the classification shown in Table 1. In the works found, a minimum of 5 participants and a maximum of 260 have been counted, having used other variables besides EDA like BVP, TMP, EEG, EOG, EMG, ECG, ACC, PUP and IBR.

A total of 21 papers have used EDA signals alone [16,17,18,19,21,22,24,25,26,42,48,49,50,51,65,74,78,79,80,132]. The use of deconvolution methods was emphasised to obtain the distinctive features of the EDA signals. Another variable that is used to help determine different emotional states in the participants is BVP, which gets particularly good results in the prediction when combined with EDA [28,32,58,59,70,75,82].

Table 4, Table 5, Table 6, Table 7, Table 8, Table 9 and Table 10 show other physiological variables used. Articles including TMP focus on its integration for stress detection. On the other hand, when adding the EMG signal, the results are slightly improved. This may be since this physiological variable complements itself very well with EDA. Another variable used for stress measurement is EEG mixed with EDA. This type of signal is widely used individually and provides good results in stress detection. Nonetheless, EEG requires very expensive and precise devices and quite specific knowledge to set up the acquisition of the signals. Finally, IBR also supplies additional information to improve the classifiers, but without achieving great improvements.

These physiological variables are excellent complements to the EDA, providing a leap in the quality of the classifier results. It is possible to supply a more realistic map of the physiological state by combining the variables. This is largely because the several variables are regulated by different systems like the SNS, the parasympathetic nervous system or a mixture of both (the autonomous nervous system).

### 4.2. Time Windows and Intervals in Arousal Detection

One aspect that has received considerable attention in this systematic review is the size of the signal segments that are used to feed each classifier. Many classifiers work better with longer signal segments and therefore more signals are introduced during the learning process. This may be due to the shape of the signal obtained, since the longer the signal, the easier it is to distinguish between the two states [105].

Regarding the minimum time for stress detection, many researchers argue that segments of at least 5 s are needed to achieve a distinction between calm and stress [26]. On the other hand, by looking at how the EDA signals are segmented, some authors use complete segments of the signals acquired in the experiments, while others prefer to use segments of EDA signals divided into smaller fragments and apply overlapping techniques to perform data augmentation and provide more data to feed the classifiers.

### 4.3. Features Most Commonly Used

Throughout the literature consulted, there is a substantial number of parameters that can be obtained from the EDA raw signals as well as from the deconvoluted signals (phasic and tonic). Due to the normalisation of data that takes place in the process, any classifier using phasic signals has a much better performance than the ones that use the raw signals.

Researchers have preferred to use time-dependent parameters more often than those based on morphology, statistics and frequency domain. Some parameters should be highlighted such as mean (Mean), numeric first and second derivative (D1, D2), standard deviations of the signal and its derivatives (SD, D1SD, D2SD), cumulative maximum (CMax) and cumulative minimum (CMin), electrodermal level (EDL) and sum rise time (SRT) or root-mean square level (RMS). The most used morphological parameters are arc length (AL), integral area (IN), normalised mean power (AP) and energy to perimeter ratio (EL). The statistical parameters used frequently are mean (M), variance (Var), median (MedVal), kurtosis (KU), skewness (SKU) and momentum (MO), in frequency domain the use of spectral power (SP), mean spectral power (MSSP) and fast Fourier transform (FFT) is quite extensive. Finally, it can be noted that Shannon entropy ($E_{Shannon}$) is one of the most widely used for time-frequency features.

### 4.4. Supervised Learning Methods

A considerable number of the papers studied use supervised learning methods (see Table 11, Table 12, Table 13, Table 14, Table 15, Table 16 and Table 17). Their main performance results are discussed below.

#### 4.4.1. Support Vector Machines

SVMs are beyond any doubt the most widely used classification methods in the papers selected. SVMs with linear, quadratic, cubic, polynomial, Gaussian, radial and radial kernels with/without PCA analysis have been proposed along the present survey.

Within arousal classification (see Table 11), SVMs with radial configuration have an F1-score and precision of 85.20% and 92.0%, respectively [15,20,28]. Furthermore, binary classifiers have an accuracy of 95.67%. In contrast, the accuracy drops to 78.93% when dealing with multi-class classification [22]. For stress classification (see Table 12), there is an F1-score and accuracy value of 92% and 90% for a deep-SVM (ensemble method) and medium-Gaussian kernel configuration, respectively [29,142].

This is closely followed by other results, also based on the radial and quadratic kernel with an accuracy rate of 83% and 81.3% for stress classification [30,45]. It is in emotion classification where the greatest number of configurations are found (see Table 13). It is also the field where the highest variability is detected. The classification results range between 63% and 91.0%, having a mean value of 79.34% accuracy [60]. In addition, it offers an accuracy of 77.6% with a radial kernel and timescale decomposition method [65] for their use in determining physical pain. Finally, the use of SVMs in oriented tasks is reinforced by results of 90.6% for a quadratic kernel and 82.7% for a radial kernel in the task-oriented group [71,75] (see Table 15).

In summary, the most used kernel, the radial kernel, obtains average results of 75.34% when all the areas of application are compared. This result achieves an acceptable performance, because other estimators such as the ROC curve or the sensitivity and specificity values are remarkably high, approaching 1 (maximum achievable level) in many cases. In addition, it should be noted that these classifiers present values higher than 90%, only comparable with the performance of the different topologies and configurations of ANNs [69] (see Section 4.4.8). Finally, when a feature reduction analysis (PCA) is applied to the previous approach, the average result of the classification is 82.24%.

#### 4.4.2. Auto-Hidden Markov Models

There are two types of algorithms within the Markov chains used for emotion and classification as shown in Table 13. On the one hand, the auto-hidden Markov chains have an associated result of 88.6% with an LDA and non-LDA approach [59]. On the other hand, there is a value of 68.7% using the standard Markov chains when considering the baseline, while the accuracy increases to 79.83% for an approach not considering the baseline [57].

#### 4.4.3. Discriminant Analysis

Discriminant analysis has been used in stress detection (see Table 12) and emotion classification (see Table 13). In this first case, the highest detection rate is 95% in accuracy for linear discriminant. As can be seen, a higher order configuration worsens the results. In contrast, the results obtained reveal an accuracy of 71.09% when applying a feature reduction algorithm to the linear discriminant. Moreover, when the discriminant employs a higher order discriminant function (quadratic or Gaussian), the results drop to 71% for stress classification. Furthermore, an accuracy of 84.7% is found in emotion classification [52]. These results suggest that the only method that can be used with acceptable results is the linear discriminant configuration. This is due to the inner workings of the classifier, as well as its ability to eliminate features that do not provide relevant information. In papers where feature removal is performed, such as in the case of LDA, something similar occurs, as will be explained below.

#### 4.4.4. Decision Trees

There are many different decision trees in the papers surveyed. Within arousal (see Table 11) and stress classification (see Table 12), random forest (RF) has been used with an accuracy of 83.58% and 91.1%, respectively [15,33] and decision tree (DT) has reached an accuracy of 96.6% [35]. Moreover, in the realm of emotion classification (see Table 13), different configurations are found with high percentages of accuracy. We have 93.5% and 80.83% accuracy for RF. For instance, we have 78.8% for the ensemble bagged method and 73.30% for the regression tree. Eventually, for classifying bodily states (see Table 17), RF is used. This technique achieves an accuracy of 73.0% using PCA analysis [80]. Lastly, in the task-oriented group (see Table 15), regression tree with 90.16% and 91.3% accuracy, using classification and regression trees (CART) and ID4-5 configurations, respectively [74], should be highlighted.

The implementation of this algorithm used the Matlab library called *”App learner”* with standard configurations (Gini criterion) in most articles selected in the systematic review.

#### 4.4.5. Naive Bayes

As for the Bayes classifier in emotion classification, the results obtained for the Gaussian configuration combined with PCA is 70.8% [61]. Generally, results with Bayes classifiers are quite poor because they assume independence in the variables (which is not the case for EDA signals).

#### 4.4.6. Logistic Regression

The use of logistic regression is not widely used in the selected papers. An accuracy of 90.19% is achieved by fusing multiple signals in stress classification [27]. On the other hand, in emotion classification an accuracy of 57.54% is obtained for a zero-regression structure [48]. Finally, for dehydration monitoring, an accuracy of 62% is obtained. Compared to others found in this study, this type of classifier is not widely used with biological signals, so the results are in line with expectations.

#### 4.4.7. K-Nearest Neighbours

KNN is one out of the most frequently adopted classifiers in physiological classification (also for EDA). The most widely used is KNN-Medium according to the reviewed literature. This type of configuration uses a not exceptionally large cluster size, which makes it more immune to noise produced by outlier data. In this sense, for arousal classification (see Table 11), the KNN-Weighted algorithm has a precision of 76.53%. Moreover, KNN-Medium can be found in stress classification with an F1-Score of 84.10% and an accuracy of 77%, respectively [27,29] (see Table 12). Moreover, the different topologies found for emotion classification (see Table 13) are KNN-Fine, KNN-Medium and KNN-Fuzzy with accuracy of 87.7%, 65.0% and 86.6% [43,46]. KNN-Cubic and KNN-Medium have obtained a precision of 87.78% and 91.2%, respectively [79,82], when monitoring dehydration (see Table 17).

#### 4.4.8. Artificial Neural Networks

The perceptron multilayer with backpropagation obtains an F1-score of 82.76% for arousal classification (see Table 11). Three distinct topologies stand out in stress classification (see Table 12), namely, ANFIS networks, recurrent networks (RNN and LSTM) and convolutional networks (CNN-LSTM) with an accuracy of 95%, 95.1% and 91.43%, respectively. Another configuration uses the novel LUCCK method (concave and convex kernel) with a result of 89.23%, in line with those obtained previously. On the other hand, multilayer perceptron is employed in emotion classification (see Table 13). This algorithm varies between 77.3% and 92.8% accuracy [23,53]. In addition, for stress classification (see Table 12), several innovative networks have been used. In this case, a Bayesian network (BPNN) and a probabilistic network (PNN) have been used, yielding results in the same range as more established networks [44,61].

Interesting in the classification of physical pain (see Table 14) is the use of the so-called late-fusion architecture topology [67]; even so, the results are a bit lower than the rest of the convolutional networks, 84.4% against 91.43%. Lastly, let us highlight the use of ANNs in the areas dedicated to monitoring. The LUCKK algorithm is used to monitor sleep and fatigue with a result of 88.3% [81] (see Table 17). In task-oriented applications (see Table 15), Adaboost achieves an accuracy of 99.69%. The three- and five-layer configurations provide a precision of 95.02% and 98.81%, respectively, for multilayer perceptron in the feedforward configuration. One-dimensional convolutional networks (1D-CNN) have also been used with results of 88.74% and 90.54%. Among the less used techniques, extreme gradient boost (XDA), adaptive neurofuzzy approach (ANFIS) and spectro-temporal ResNet have shown results of 94%, 76.7% and 80.0% precision, respectively.

#### 4.4.9. Long Short-Term Memory and Recurrent Neural Networks

In the domain of stress classification, attending to the different configurations, LSTM may be used alone or in other configurations through assembly method. For an LSTM network, the F1-score is 81.4%, while the CNN + LSTM obtains an F1-score of 79.13%. The ANFIS configuration variant gets 95%. Although there is little literature on this type of classifier, it should be regarded as a suitable alternative when using a dataset in the time domain based on the processed electrodermal activity response (SCR).

### 4.5. Unsupervised Learning Methods

There is truly little literature regarding unsupervised learning methods (see Table 18). Below are the most used methods studied throughout this review and their most important results.

One of the unsupervised learning algorithms used is K-means. This algorithm achieves a precision of 77.5%. The K-medoids approach has also been evaluated to minimise the effects of noise produced in outlier data on a dataset. The result of 75.5% precision is at the same level as those obtained for K-means. Finally, as an alternative method to the previous ones, there are the methods based on self-organising maps (SOMs) within the unsupervised learning techniques. In this case, the results obtained for this classifier are at the same level as the earlier ones (77.5%).

## 5. Conclusions

This paper has presented a systematic review on the use of physiological signals for arousal detection and classification, focusing on electrodermal activity (EDA) and various machine learning techniques. At first, a total of 228 papers were considered, of which fifty-nine were selected for the in-depth systematic review. These articles provided a global perspective on a specific topic such as the use of EDA, individually or in conjunction with other variables, for the classification of arousal categorisations and related terms using ML techniques.

One aspect that has emerged during this review is the different groups of applications or categorisations found in the search for terms related to arousal detection. The following categories were found: stress detection, emotion classification, physical pain affectation, task-oriented performance, mental/cognitive workload estimation and, finally, a small group of specific applications such as sleep monitoring and dehydration.

Several critical issues have arisen throughout this study that should be kept in mind by researchers interested in signal acquisition in general and EDA processing in particular. The first point to consider is that the classification process must be addressed from the moment the signals are obtained (acquisition process). The signals become useless for further classification without a robust acquisition process. In addition, most of the authors studied in this systematic review underline that this process is not exempt from dealing with signal interference, artefacts and noise. A proper application of the different filters during the pre-processing stage becomes crucial for the following phases. All articles studied on EDA signals emphasise that the signals must go through a deconvolution process for homogenisation and normalisation. The normalisation process makes it possible to use a dataset that has a large amount of data without being affected by race, sex and age. In fact, studies in which there was no deconvolution process have been discarded because of the poor results obtained with any classifier.

Once the signals have been pre-processed, the next important step is to obtain distinctive features. Most authors agree on using different domains, usually the time domain and the frequency domain or a mixture of both in the time-frequency domain. There are also approaches that analyse the shape of the signal (morphological) and others that analyse the signal statistically (statistical features). No one agrees on the number of variables or the minimum number of functions to be used. The general approach is to use several types and fit the model by LDA, PCA or ICA analysis to perform dimensionality reduction.

In addition, two distinct methods have been found for estimating the participant’s emotional state during the review. The first approach aims to use only EDA for detection, while the second is to use EDA signals complemented by other physiological signals such as BVP, ECG and EMG, among others. One of the advantages of using EDA alone is the possibility of incorporating small, non-invasive devices with high autonomy. Another advantage is that the results using only EDA are quite good. In contrast, using more physiological signals offers the advantage of monitoring several types of responses, which provides a better mapping of the subject’s physical, psychological and cognitive state. However, a disadvantage is that the use of different signals makes the system more complex and more difficult to maintain and causes it to have a higher classification computational cost.

Although EDA is a particularly good indicator for the detection of arousal changes in the individual, it has its limitations. As an SNS-dependent variable, several different stimuli can be detected as arousal changes. This is why it should be preferred to use with other physiological signals such as the BVP, among others. Combining the EDA with these signals makes the results more reliable as they respond to various parts of the nervous system.

When considering the classification methods found, most authors favour the use of techniques based on supervised learning. This is largely because the experiments and datasets are labelled for each of the states. For this reason, few articles use unsupervised techniques. Among the supervised learning methods, SVMs and many of the ANN topologies show the best classification results, closely followed by KNN algorithms. For SVMs, those implementing quadratic, cubic and radial kernels outperform with accuracy 85.26%, 82.86% and 82.4%, respectively. ANNs, on the other hand, highlight for their accuracy in different configurations, especially ANN-Adaboost with 99% and different configurations of the multilayer perceptron with 95% and 98% for the three-layer and five-layer sorts, respectively.

The above results would be biased by only looking at the overall results of the classifiers, because the papers used different datasets and experimental conditions. Therefore, we have indicated which classifiers are predominant for each arousal detection category. The most common classifier found is the SVM in the arousal variation detection group. For stress detection, the most used classifier is ANN, closely followed by SVM. The same holds for emotion detection and classification. Similarly, there is a tie between SVM and ANN in the detection and estimation of physical pain. Finally, there is a mix of KNN, SVM, BPNN, LDA and decision trees in the detection of cognitive/mental load, as well as in the rest of the groups.

Our aim has been to acquaint the researcher with the methods of acquiring, processing and extracting features and classifying EDA signals. This gives an overview of the work to be done and the methods that work or do not work successfully. As a conclusion we can state that the use of EDA alone for the detection (and subsequent classification) of arousal is very widespread and very satisfactory results have been achieved. Moreover, its use in combination with other physiological signals and with the help of robust and novel ML techniques has been growing over time. For this reason, arousal classification is being integrated non-invasively into user-centred devices, while at the same time the robustness and accuracy of current systems and applications have been enhanced.

## Figures and Tables

**Table 1:** Paper classification by group.

Arousal	[15,16,17,18,19,20,21,22,23]
Stress	[24,25,26,27,28,29,30,31,32,33,34,35,36,37,38,39,40,41]
Emotion	[42,43,44,45,46,47,48,49,50,51,52,53,54,55,56,57,58,59,60,61,62,63,64]
Physical Pain	[65,66,67,68]
Task-Oriented	[69,70,71,72,73,74,75]
Mental Workload	[70,76,77]
Others	[78,79,80,81,82,83,84,85,86,87]

**Table 2:** Bio-signals and their properties.

Signal	Abbrev.	Ch.	SF (Hz)	RF (Hz)	AL
Electrocardiogram	ECG	1–12	0.05–150	250–1K	0.1–5
Electromyography	EMG	1–32	25–5K	512–10K	0.1–100
Blood Volume Pressure	BVP	1	0.25–40	5–500	−10–10
Electrooculography	EOG	2	0–100	1–100	50–3.5K
Pupillography	PUP	2	120	240	-
Electroencephalography	EEG	1–128	128–2K	128–2K	1–150 mV
Inter-Breath	IBR	1	1–20	1–20	−0.05–0.05
Acceleration	Acc	3	20–2K	20–2K	−1–1
Skin Temperature	TMP	1	1–200	2–50K	−50–50
Electrodermal Activity	EDA	1	1–16	16–128	0–100 μS

Significance Frequency (SF), Channel (Ch), Record Frequency (RF), Amplitude (AL).

**Table 3:** Features obtained in the process.

Domain	Features
Time	Mean *, SD*, D1*, D2 *, D1M *, D2M *, D1SD *, D2SD *, EDL *
	SRT *, SFT, RM, RRSTD, DCRM, DCRSD, RM, PHVM, PHVSD,
	RRSTD, DCRM, DCRSD, STM, STSD, STRMS, STRMSSD
	STRMSOV, EDL, EDR, CMax *, CMin *, SWE, DR, RMS *,
	PMRMSR, RSSL, P, PLoc, PPT, pNN50 *
Morphological	NO, EC, EP, EPC, EN, AL *, IN *, AP *, RMS *, IL *, EL *
Statistical	M *, Var *, MedVal *, p-Val, AKAIKE, LOG-LIKE, FCM, FVCM
	KU *, SKU *, MO *, COVMAT
Frequency	SP *, SSP, MSSP *, SSPMed, NSSCRs, FFT *, PSD
Time-Frequency	$TF_{Flux}$, $TF_{Flatness}$, $TF_{Energy}$, TVSymp, MFCC, $E_{Shannon}^{*}$, $E_{Log}$

Note: * most used features.

**Table 4:** Physiological Signals Used for Arousal Detection.

Papers	Year	Parameters	Participants	Evaluation	Annotations
Chowdhury et al. [15]	2019	EDA BVP TMP	22	F-score + ML	
Greco et al. [16,17,18]	2014–2019	EDA	18–32	ML Met.	
Kelsey et al. [21]	2018	EDA	73	ML Met.	
Khalaf et al. [19]	2020	EDA	260	ML Met.	Clustering maps
Kleckner et al. [20]	2018	EDA TMP	20	ML Met.	
Taylor et al. [22]	2015	EDA ECG	100	ML Met.	Wavelet transform
Zhang et al. [23]	2017	EDA BVP TMP	87	ML Met.	

**Table 5:** Physiological signals used for stress detection.

Papers	Year	Parameters	Participants	Evaluation	Annotations
Anusha et al. [24]	2017	EDA	12	ML Met.	Stressors in EDA
Anusha et al. [25]	2020	EDA	41	ML Met.	Pre-Surgery stress EDA
Aristizabal et al. [41] Cho et al. [28]	2017	EDA BVP	12	ML Met.	Unsupervised Learning
Hadi et al. [33]	2019	EDA BVP IBR EMG	59	ML Met.	SVM-RBF best perf.
Jebelli et al. [29]	2019	EDA BVP TMP	10	ML Met.	Stress in workers
Liapis et al. [38]	2021	EDA SKT	–	ML Met.	SVM models
Lee. et al. [40]	2021	EDA		ML Met.	CNN networks
Martinez et al. [35]	2019	EDA BVP IBR	18	ML Met.	Expert system
Nath et al. [37]	2021	EDA + BVP	41	ML Met.	RF, SVM and LR
Rastgoo et al. [34]	2019	EDA ECG	6	ML Met.	LSTM model
Sanchez-Reolid [26]	2020	EDA	147	ML Met.	D-SVM based
Setz et al. [30]	2010	EDA EMG	33	ML Met.	Stress cognitive
Siddarth et al. [31]	2020	EDA BVP EEG	12	ML Met.	LSTM model
Singh et al. [32]	2013	EDA BVP	19	ML Met.	NN topologies
Wang et al. [39]	2021	EDA	–	ML Met.	Ensemble ANN methods
Zontone et al. [36]	2022	EDA+ECG	18	ML Met.	SVM classifier

**Table 6:** Physiological signals used for emotion detection.

Papers	Year	Parameters	Participants	Evaluation	Annotations
Al-Machot et al. [42]	2018	EDA ECG	30	SAM’s + ML	MAHNOB dataset
Al-Machot et al. [43]	2019	EDA BVP EMG IBR	30	SAM’s + ML	MAHNOB dataset
Ali et al. [44]	2018	EDA BVP TMP	30	ML Met.	MAHNOB dataset
Anderson et al. [45]	2017	EDA BVP EOG	41	ML Met.	Multi-class classifier
Cavallo et al. [46]	2019	EDA BVP EEG	34	ML Met.	Multi-class model
Fiorini et al. [47]	2020	EDA BVP IBR	50	SAM + ML	
Ganapathy et al. [49]	2020	EDA	32	ML Met.	Convolutional Analysis
Ganapathy et al. [62]	2021	EDA	32	ML Met.	CNN multi-scale
Garcia-Faura et al. [48]	2019	EDA	14	ML Met.	
Greco et al. [50,51]	2014–2019	EDA	18–32	ML Met.	
Jang et al. [52]	2015	EDA	40	ML Met.	
Katsis et al. [73]	2008	EDA BVP IBR EMG	20	ML Met.	Automatic method
Katsis et al. [53]	2011	EDA BVP IBR	5	ML Met.	Multi-class classification
Khezri et al. [54]	2015	EDA BVP IBR EMG	20	ML Met.	
Kim et al. [55]	2018	EDA BVP EEG	30	ML Met.	
Kukolja et al. [56]	2014	EDA BVP	14	ML Met.	
Liu et al. [132]	2019	EDA	21	ML Met.	Kappa coefficients
Liu et al. [57]	2019	EDA BVP EMG	17	Accuracy	Markov-Chain Based
Pinto et al. [58]	2019	EDA BVP	23	ML Met.	Multi-class classifier
Rajendran et al. [64]	2022	EDA BVP		ML Met.	Recurrent NN
Zhang et al. [60]	2017	EDA ACC	87	ML Met.	Unsupervised ML
Zhao et al. [61]	2018	EDA BVP TMP	32	ML Met.	PCA analysis
Zontone et al. [75]	2020	EDA BVP	18	ML Met.	

**Table 7:** Physiological signal used for physical pain detection.

Papers	Year	Parameters	Participants	Evaluation	Annotations
Kong et al. [68]	2021	EDA	10	ML Met.	Pain using Heat
Susam et al. [65]	2018	EDA	34	ML Met.	
Thiam et al. [67]	2019	EDA BVP EMG	87	ML Met.	BioVid Database
Walter et al. [66]	2013	EDA ECG EMG EEG	90	Statistical	BioVid Heat Pain Dataset

**Table 8:** Physiological signals used in task-oriented experiments.

Papers	Year	Parameters	Participants	Evaluation	Annotations
Bianco et al. [69]	2019	EDA BVP IBR	68	ML Met.	Deep classifier
Ding et al. [70]	2020	EDA	35	ANOVA + ML	
Gjoreski et al. [72]	2020	EDA EOG PUPIL	68	ML Met.	
Momin et al. [74]	2019	EDA	–	ML Met.	Task-oriented

**Table 9:** Physiological signals used for mental/cognitive workload detection.

Papers	Year	Parameters	Participants	Evaluation	Annotations
Ding et al. [70]	2020	EDA	18	MLT Met.	Simulated computed task
Jimenez-Molina et al. [76]	2018	EDA BVP EEG	61	MLT Met.	Web browsing workload
Lanata et al. [77]	2017	EDA IBR ECG	15	MLT Met.	Driving monitoring

**Table 10:** Physiological signals used for other physical states detection.

Papers	Year	Parameters	Participants	Evaluation	Annotations
Amidei et al. [87]	2022	EDA	9	ML Met.	Driver drowsiness
Chowdhury et al. [140]	2022	EDA ACC	12	ML Met.	Epileptic seizure detection
Hwang et al. [78]	2017	EDA	17	ML Met.	Sleep Monitoring
Hossain et al [84]	2022	EDA	20	ML Met.	Artifact detection
Rizwan et al. [79]	2020	EDA	5	ML Met.	Dehydration Detection
Posada-Quintero [82]	2019	EDA ECG	70	ML Met.	Dehydration Detection
Sadeghi et al. [80]	2020	EDA	41	ML Met.	Sleep Monitoring
Sabeti et al. [81]	2019	EDA BVP ACC TMP	20	LUCKK	Sleep Monitoring
Sandeghi et al. [80]	2019	EDA BVP ACC	20	ML Met.	Sleep Monitoring
Yin. G. et al. [83]	2022	EDA	32	ML Met.	Residual Neural Networks

**Table 11:** Supervised learning methods for arousal classification.

Authors	MLT	Type	Conf.	Performance *	Annotations
Chowdhury et al. [15]	SL	SVM	Radial (RBF)	$85.20\left(0\right){\;}^{3}$	EDA +HR +TMP fusion
	SL	TREE	RF	$83.58\left(0\right){\;}^{3}$	EDA +HR +TMP fusion
	SL	ANN	MLP-BP	$82.76\left(0\right){\;}^{3}$	EDA +HR +TMP fusion
Greco et al. [16,17,18]	SL	SVM	Radial (RBF)	$69.9\left(0\right){\;}^{1}$	EDA + HRV
Khalaf et al. [19]	SL	SVM	Radial (RBF)	$76.46\left(0\right){\;}^{1}$	
Kleckner et al. [20]	SL	SVM	–	$92.0\left(0\right){\;}^{1}$	Cohen’s κ=0.55
Taylor et al. [22]	SL	SVM	Radial (RBF)	$95.67\left(0\right){\;}^{1}$	Binary Artefact detection
	SL	SVM	Radial (RBF)	$78.93\left(0\right){\;}^{1}$	Multi-class Artifact detection
Zhang et al. [23]	SL	KNN	Weighted	$76.53\left(8.64\right){\;}^{2}$	ML Met.

Note: 1 = accuracy; 2 = precision; 3 = F1-score.; * Mean performance and its standard deviation.

**Table 12:** Supervised learning methods for stress classification.

Authors	MLT	Type	Config	Performance *	Annotations
Anusha et al. [24]	SL	DISC.	Linear	$95.1\left(0\right){\;}^{1}$	
Anusha et al. [25]	SL	DISC.	PCA + LDA	$71.09\left(0\right){\;}^{1}$	PCA analysis
	SL	ANN	ANFIS	$95\left(0\right){\;}^{2}$	ANFIS-Based Short-Term
Sanchez-Reolid [26]	SL	SVM	Radial	$83.0\left(0\right){\;}^{3}$	
	SL	SVM	Deep-SVM	$92.0\left(0\right){\;}^{3}$	Deep-SVM ensemble
Can et al. [27]	SL	ANN	MLP	$92.15\left(0\right){\;}^{3}$	HR + EDA + ACC
	SL	Logistic reg.	Standard	$90.19\left(0\right){\;}^{3}$	HR + EDA + ACC
	SL	KNN	–	$84.10\left(0\right){\;}^{3}$	HR + EDA + ACC
Cho et al. [28]	SL	ANN	K-ELM	$95.1\left(0\right){\;}^{2}$	Feed-forward NN (SLFNs)
Jebelli et al. [29]	SL	SVM	Medium-Gauss.	$90\left(0\right){\;}^{1}$	
	SL	DISC.	GDA	$71\left(0\right){\;}^{1}$	Gaussian DA
	SL	KNN.	Medium	$77\left(0\right){\;}^{1}$	
Setz et al. [30]	SL	SVM	Quadratic	$81.3\left(0\right){\;}^{1}$	
	SL	DISC.	Linear	$82.8\left(0\right){\;}^{1}$	
Siddarth et al. [31]	SL	ANN	CNN-LSTM	$91.43\left(5.17\right){\;}^{1}$	VGG-16 Net + PCA + LSTM
Singh et al. [32]	SL	ANN	LUCCK	$89.23\left(0\right){\;}^{2}$	Concave and Convex Kernel
	SL	ANN	LRNN	$89.23\left(0\right){\;}^{2}$	Recurrent NN
Hadi et al. [33]	SL	TREE	RF	$91.1\left(0\right){\;}^{1}$	
Rastgoo et al. [34]	SL	LSTM	CNN + LSTM	$79.13\left(2.47\right){\;}^{3}$	Ensemble CNN + LSTM
	SL	LSTM	LSTM	$81.4\left(0\right){\;}^{3}$	
Martinez et al. [35]	SL	TREE	DT	$96.6\left(0\right){\;}^{1}$	Decision tree algorithm

Note: 1 = accuracy; 2 = precision; 3 = F1-score.; * Mean performance and its standard deviation.

**Table 13:** Supervised learning methods for emotion classification.

Authors	MLT	Type	Config	Performance *	Annotations
Al-Machot et al. [42]	SL	ANN	CNN	$82\left(0\right){\;}^{1}$	MAHNOB dataset
Al-Machot et al. [43]	SL	SVM	Radial	$63.0\left(0\right){\;}^{2}$	Matlab + ML Met.
	SL	KNN	Medium (k = 3)	$65\left(0\right){\;}^{2}$	Matlab + ML Met.
Ali et al. [44]	SL	ANN	MLP-BP	$80.0\left(0\right){\;}^{3}$	NN based.
	SL	BPNN	Bayes	$89.38\left(0\right){\;}^{3}$	Cellular-NN
Anderson et al. [45]	SL	SVM	Medium-Gauss.	$83.3\left(0\right){\;}^{3}$	Matlab + ML Met.
	SL	TREE	Bagged	$78.8\left(0\right){\;}^{3}$	Matlab + ML Met.
Cavallo et al. [46]	SL	SVM	Quadratic	$89.67\left(0\right){\;}^{3}$	Matlab + ML Met.
	SL	SVM	Radial + PCA	$82.4\left(0\right){\;}^{3}$	Matlab + ML Met.
	SL	KNN	Fuzzy	$86.6\left(0\right){\;}^{3}$	Matlab + ML Met.
	SL	KNN	Fine	$87.7\left(0\right){\;}^{3}$	Matlab + ML Met.
Fiorini et al. [47]	UL	K-means	Standard	77.5(2.12)	Standard config.
	UL	K-medoids	Standard	75.5(2.12)	Standard config.
	UL	SOM	Standard	77.5(0.5)	Bi-dimensional map
Garcia-Faura et al. [48]	SL	Logistic Reg.	ZeroR	$57.54\left(0\right){\;}^{2}$	Zero Regression
Ganapathy et al. [49]	SL	CNN	MLP-BP	$71.41\left(0\right){\;}^{3}$	NN based.
Jang et al. [52]	SL	DISC.	DFA	$84.7\left(0\right){\;}^{1}$	Discriminant analysis
Katsis et al. [53]	SL	SVM	Radial (RBF)	$78.5\left(0\right){\;}^{1}$	10s + 5 emotions
	SL	TREE	RF	$80.83\left(0\right){\;}^{1}$	10s + 5 emotions
	SL	ANN	MLP	$77.33\left(0\right){\;}^{1}$	10 s + 5 emotions
	SL	NFS	Fuzzy Inference	$84.3\left(0\right){\;}^{1}$	10 s + 5 emotions
Khezri et al. [54]	SL	SVM	Radial	$82.7\left(0\right){\;}^{1}$	
Kim et al. [55]	SL	SVM	Radial	$74\left(0\right){\;}^{1}$	
Kukolja et al. [56]	SL	ANN	MLP-BP	$60.30\left(0\right){\;}^{1}$	Baseline EDA
Liu et al. [57]	SL	Markov	Markov-Chain	$68.74\left(7.85\right){\;}^{1}$	With Baseline
	SL	Markov	Markov-Chain	$79.83\left(5.67\right){\;}^{1}$	Without Baseline
Pinto et al. [58]	SL	SVM	Radial	$69.13\left(0\right){\;}^{1}$	
Patlar et al. [59]	SL	Markov	Auto-Hidden	$88.6\left(0\right){\;}^{1}$	With LDA + Acc.
	SL	Markov	Auto-Hidden	$86.6\left(0\right){\;}^{1}$	Without LDA +Acc.
Rajendran et al. [64]	SL	LSTM		$99.0\left(0\right){\;}^{1}$	
Zhang et al. [60]	SL	SVM	Radial	$91.4\left(0\right){\;}^{1}$	Motion Artifact
	SL	TREE	RF	$93.5\left(0\right){\;}^{1}$	Motion Artifact
	SL	ANN	MLP-BP	$92.8\left(0\right){\;}^{1}$	Motion Artifact
Zhao et al. [61]	SL	TREE	Regression	$73.30\left(2.99\right){\;}^{2}$	Matlab + ML Met.
	SL	Naïve-Bayes	Gaussian	$70.8\left(0.53\right){\;}^{1}$	PCA analysis
	SL	PNN	Probabilistic	$71.31\left(0\right){\;}^{3}$	Probabilistic NN

Note: 1 = accuracy; 2 = precision; 3 = F1-score.; * Mean performance and its standard deviation.

**Table 14:** Supervised learning methods for physical pain classification.

Authors	MLT	Type	Config	Performance *	Annotations
Susam et al. [65]	SL	SVM	Radial	$77.6\left(0\right){\;}^{1}$	Timescale decomposition (TSD)
Thiam et al. [67]	SL	ANN	CNN-DL	$84.40\left(14.43\right){\;}^{1}$	Convolutional + Late fusion architecture

Note: 1 = accuracy; 2 = precision; 3 = F1-score; * Mean performance and its standard deviation.

**Table 15:** Supervised learning methods for task-oriented applications.

Authors	MLT	Type	Config	Performance *	Annotations
Bianco et al. [69]	SL	ANN	1D-CNN	$88.74\left(0\right){\;}^{3}$	Convolutional-NN
	SL	ANN	1D-CNN-E	$90.54\left(0\right){\;}^{3}$	Convolutional ensemble
	SL	ANN	Adaboost	$99.69\left(0\right){\;}^{1}$	Adaboost Method
	SL	ANN	3-NN	$95.02\left(6.34\right){\;}^{2}$	
	SL	ANN	5-NN	$98.81\left(0\right){\;}^{2}$	
Ding et al. [70]	SL	ANN	1D-CNN	$96.4\left(0\right){\;}^{1}$	Convolutional-NN
Gharderyan et al. [71]	SL	SVM	Quadratic	$90.6\left(0\right){\;}^{1}$	Wavelet + features
	SL	CNN	MLP-BP	$80.2\left(0\right){\;}^{1}$	NN based
Gjoreski et al. [72]	SL	ANN	XDA	$94.0\left(0\right){\;}^{3}$	Extreme Gradient Boost
	SL	ANN	CNN-LSTM	$75\left(0\right){\;}^{3}$	
	SL	ANN	STR-Net	$80\left(0\right){\;}^{3}$	SpectroTemporal-ResNet
Katsis et al. [73]	SL	SVM	Radial	$79.3\left(0\right){\;}^{1}$	
	SL	ANN	ANFIS	$76.7\left(0\right){\;}^{1}$	Adaptive Neuro-Fuzzy
Momin et al. [74]	SL	SVM	Radial	$82.7\left(8.9\right){\;}^{1}$	
	SL	TREE	Regression	$90.16\left(4.65\right){\;}^{1}$	CART config.
	SL	TREE	DT	$91.3\left(0\right){\;}^{1}$	ID4-5 config.
Posada-Quintero et al. [141]	SL	KNN	Medium	$66.0\left(0\right){\;}^{1}$	
Zontone et al. [75]	SL	SVM	Radial	$76.72\left(0\right){\;}^{1}$	
	SL	ANN	MLP	$77.15\left(0\right){\;}^{1}$	

Note: 1 = accuracy; 2 = precision; 3 = F1-score.; * Mean performance and its standard deviation.

**Table 16:** Supervised learning methods for classification of mental/cognitive workload.

Authors	MLT	Type	Config	Performance *	Annotations
Ding et al. [70]	SL	BPNN	Bayesian	$77.80\left(0\right){\;}^{1}$	Only physiological
	SL	SVM	Cubic	$76.33\left(0\right){\;}^{1}$	Only physiological
	SL	KNN	Weighted	$75.67\left(0\right){\;}^{1}$	Only physiological
	SL	Tree	Fine	$73.33\left(0\right){\;}^{1}$	Only physiological
	SL	LDA	–	$61\left(0\right){\;}^{1}$	Only physiological
Jimenez-Molina et al. [76]	SL	ANN	MLP	$93.7\left(0\right){\;}^{1}$	Combined EDA+EEG+BVP
Lanata et al. [77]	SL	MNC	–	$91\left(0\right){\;}^{1}$	MNC model

Note: 1 = accuracy; 2 = precision; 3 = F1-score; * Mean performance and its standard deviation.

**Table 17:** Supervised learning methods for classification of other states.

Authors	MLT	Type	Config	Performance *	Annotations
Amidei et al [87]	SL	RF	RF	$84.1\left(0\right){\;}^{1}$	Driver drowsiness
Chowdhury et al. [140]	SL	SVM	Rbf	86.9	Driver drowsiness
	SL	DT	Bagged	90.7	Driver drowsiness
Hwang et al. [78]	SL	Disc.	–	$65.0\left(0\right){\;}^{2}$	Sleep time algorithm
Rizwan et al. [79]	SL	KNN	Medium	$87.78\left(0\right){\;}^{1}$	Dehydration
	SL	Logistic Reg.	Standard	$62.0\left(0\right){\;}^{1}$	Dehydration
Sadeghi et al. [80]	SL	TREE	RF	$73.0\left(0.53\right){\;}^{1}$	PCA analysis
Sabeti et al. [81]	SL	ANN	LUCCK	$88.38\left(5.55\right){\;}^{1}$	LUCCK Config.
Posada-Quintero et al. [82]	SL	KNN	Cubic	$91.2\left(0\right){\;}^{1}$	Dehydration

Note: 1 = accuracy; 2 = precision; 3 = F1-score; * Mean performance and its standard deviation.

**Table 18:** Unsupervised learning methods for emotion classification.

Group	Type	Config.	Papers	Precision *	Annotations
Emotion	K-means	Standard	[47]	77.5(2.12)	Standard configuration
Emotion	K-medoids	Standard	[47]	75.5(2.12)	Standard configuration
Emotion	SOM	Standard	[47,52]	77.5(0.5)	Bi-dimensional map

Note: * Mean performance and its standard deviation.

## Data Availability

Not applicable.

## Abbreviations

The following abbreviations are used in this manuscript:

ACC	Acceleration
ACR	Accuracy
AHMM	Auto-hidden Markov Model
AI	Artificial Intelligence
AKAIKE	AKAIKE Information Criterion
AL	arc length
ANNs	Artificial Neural Networks
AP	normalised mean power
AR-HMM	Auto-Regressive Hidden-Markov Model
AUC	Area Under Curve
BioVid	BioVid Heat Pain Database
BPNN	Bayesian Probabilistic Neural Network
BVP	Blood Volume Pressure
CMax	Cumulative Maximum
CMin	Cumulative Minimum
CNN	Convolutional Neural Network
COVMAT	Covariance Matrix
D1	First Derivative
D1M	First Derivative Mean
D1SD	First Derivative Standard Deviation
D2	Second Derivative
D2M	Second Derivative Mean
D2SD	Second Derivative Standard Deviation
DA	Discriminant Analysis
DCRM	Decay Rate Mean
DCRSD	Decay Rate Standard Deviation
DEAP	Database for Emotion Analysis using Physiological Signal
DR	Dynamic Range
DTs	Decision Trees
EC	Epoch-Capacity
ECG	Electrocardiogram
EDA	Electrodermal Activity
EDL	Electrodermal Level
EDR	Electrodermal Response
EEG	Electroencephalography
EL	Energy to Perimeter Ratio
EMG	Electromyography
EN	Entropy
EOG	Electrooculography
EP	Epoch-Peak
EPC	Epoch Peak Counter
FCM	Four Central Moment
FFT	Fast Fourier Transform
FVCM	Five Central Moment
GDA	Gaussian Discriminant Analysis
IBR	Inter-Breath
IL	Perimeter to Area Ratio
IN	Integral Area
IRF	Impulse Response Function
KNN	K-nearest Neighbours
KU	Kurtosis
LDA	Linear Discriminant Analysis
LOG-LIKE	Log-likelihood
LR	Logistic Regression
LSTM	Long Short-Term Memory
MAHNOB	Multi-modal Data base for Affect Recognition
MAt	Motion Artefact
Mean	Mean
Median-Val	Median Value
MedVal	Median Value
ML	Machine Learning
MLP	Multilayer Perceptron
MLT	Machine Learning Techniques
MO	Momentum
MSSP	Mean Spectral Components
NO	Number of Observation
NSSCRs	Frequency Non-Specific of Skin Conductance Response
P	Peak
p-Val	*p*-value
PHVM	Phasic Value Mean
PHVSD	Phasic Value Standard Deviation
PLoc	Peak Location
PMRMSR	Peak-Magnitude-to-RMS Ratio
pNN50	Peaks Intervals Differs 50 ms
PPT	Peak to Peak Time
PUP	Pupillometry
QDA	Quadratic Discriminant Analysis
RM	Rise Rate Mean
RMS	Root-mean Square Level
RNN	Recurrent Neural Network
ROC	Receiver Operating Characteristics
RRSTD	Rise Rate Standard Deviation
RSSL	Root Sum of Squares Level
SC	Skin Conductance
SCL	Skin conductance Level
SCR	Skin Conductance Response
SD	Standard Deviation
SFT	Sum Fall Time
SKU	Skewness
SOM	Self-Organising Maps
SP	Spectral Power
SRT	Sum Rise Time
SSP	Sum Spectral Power
SSPMed	Median Spectral Power Components
STM	Startle Time Mean
STRMS	Startle Time Mean
STRMSOV	Startle RMS Overall
STRMSSD	Startle RMS Standard Deviation
STSD	Startle Time Standard Deviation
SVM	Support Vector Machine
SWE	Smallest Window Elements
TMP	Temperature
Var	Variance
